# Registered nurses’ management of depression in general wards

**DOI:** 10.4102/hsag.v28i0.2328

**Published:** 2023-11-28

**Authors:** Mandisa Mpetshu, Jeanette E. Maritz

**Affiliations:** 1 Department of Health Studies, Faculty of Human Sciences, University of South Africa, Pretoria, South Africa

**Keywords:** COVID-19 pandemic, depression, death anxiety, general medical ward, patients, nurses

## Abstract

**Background:**

During and in the aftermath of the COVID-19, the rate of depression increased globally. A significant number of patients found in a general hospital or ward with physical conditions often have depression.

**Aim:**

This study aimed to gain an in-depth understanding of registered nurses’ experiences managing patients with depression in a general medical ward.

**Setting:**

The study took place in two general medical wards of a private hospital in the Gauteng province, South Africa, in 2021, with COVID-19 lockdown levels three to one prevailing.

**Methods:**

A descriptive qualitative design was used, and data were collected through 10 in-depth, face-to-face interviews. Data were analysed using a thematic approach.

**Results:**

The COVID-19 pandemic exerted a bidirectional influence, affecting both patients diagnosed with depression while admitted to a general ward and the nurses caring for them. This mutual impact added an additional layer of complexity to patient management.

**Conclusion:**

For optimal care of patients with depression in general wards, nurses need comprehensive training, confidence and a safe environment, bolstered by sufficient resources and robust management support.

**Contribution:**

The study highlights critical challenges in detecting and caring for patients diagnosed with depression in a general medical ward and the compounding effect of COVID-19. These findings underscore the importance of addressing clinical and psychosocial needs in a healthcare setting, especially amid a global pandemic.

## Introduction

In the aftermath of the COVID-19 pandemic, a surge in mental health problems has emerged as a significant public health challenge, requiring urgent resolution (Moreno et al. 2020:813). Depression, with its characteristic mood swings, sense of worthlessness and loss of life interest, can significantly alter individuals’ eating patterns, sleep cycles, mood, overall outlook on life and productivity (American Psychiatric Organization 2021; Kraus et al. 2019:127). The National Confidential Enquiry into Patient Outcome and Death (NCEPOD 2017) reveals that patients in general hospitals often suffer from depression along with their physical ailments. They can either present with depression on admission or develop depression while hospitalised (Pannick et al. 2019:3).

Dispelling the notion that patients with depression are confined to psychiatric institutions, registered nurses in general medical wards often find themselves caring for patients diagnosed with depression, frequently accompanied by severe physical illnesses (Davis & Lockhart 2017:26; NCEPOD 2017; Walker et al. 2018:2285). When faced with a patient with depression, it is paramount that nurses have the requisite skills and self-assurance to provide comprehensive care (Harris 2018:2). However, there is often a shortfall in the hospital systems in terms of providing adequate training and support to nurses caring for patients who are depressed while admitted to a general medical ward. This lack of support can undermine nurses’ confidence in caring for such patients (Harris 2018:4).

Registered nurses’ experiences in managing patients with depression in general medical wards are, however, still not well understood (Ni et al. 2020:4). If left unaddressed, patients with depression in general medical wards could continue to be underserved. Given the profound impact of depression on individuals and society, there is a pressing need for innovative approaches to its prevention and management (Schuch & Stubbs 2019:300). This study was conducted to gain an in-depth understanding of registered nurses’ experiences in managing patients with depression in a general medical ward by asking the question, ‘what are registered nurses’ experiences managing patients diagnosed with depression in a general medical ward?’ We, therefore, explored and described nurses’ experiences managing patients diagnosed with depression in a general medical ward.

### Prevalence of depression

The rate of depression has increased globally, making visible notable shortcomings in the mental health system (Dardas 2022:3). Winkler et al. (2020:e173) suggested that a rise in mental health problems creates an additional weight for mental health stability and causes difficulty in closing existing intervals in managing patients with mental health challenges. The World Health Organization (WHO 2021) reports depression as a significant cause of unfitness worldwide and contributes to the overall ailing disease burden. Depressive disorders, such as depression, are among the most critical public health concerns as they have become the leading disease burden and disability globally and in South Africa (Nglazi et al. 2016:46). Depression is a common disease condition in the world, with more than 280 million people affected (WHO 2020). Nglazi et al. (2016:48) found that depression is among the top three causes of disease burden worldwide. Nguse and Wassenaar (2021:305) highlighted that in South Africa, mental disorders had been overlooked by the health system, with one person out of six being diagnosed with depression, anxiety or a substance use disorder, and 41% of South Africans living with HIV have depressions comorbidity, 41% of women are depressed during their pregnancy and 60% of South Africans are subject to post-traumatic stress. Only about 27% of South Africans diagnosed with mental disorders receive treatment.

COVID-19 caused by the severe acute respiratory syndrome coronavirus 2 (SARS-CoV-2) virus, was initially recognised for its severe impact on the respiratory system but soon revealed effects on various organs because of its ability to bind to the angiotensin-converting enzyme (ACE2) receptor present in numerous tissues, including the central nervous system (WHO 2020). This interaction could result in neuropsychiatric symptoms like anxiety, memory and concentration issues and depression, either concurrently with respiratory symptoms or post-recovery, with a higher incidence in hospitalised patients (Da Silva Lopes et al. 2021:1119). The global prevalence of anxiety and depression surged by 25% by the end of the pandemic’s first year, a statistics contested as possibly reflecting a short-term reaction (Daly & Robinson 2022:518; WHO 2022). Depression is multifactorial, with contributing factors like stress, isolation, fear of death and the overwhelming additional tensions introduced by the pandemic, such as unemployment and financial insecurity (Halaris 2019:422; Shader 2020:965). Demographically, women, young people and those with pre-existing conditions were more susceptible to mental health challenges (WHO 2022). Notably, those who contracted COVID-19 demonstrated a doubled risk of depression diagnosis, with many experiencing depression for the first time post-recovery (Taquet et al. 2021:135).

## Research methods and design

### Study design

This study followed a descriptive qualitative design (Creswell & Creswell 2022:196) to provide a broad and in-depth view of the area of enquiry. Qualitative research is a social inquiry that focuses on how human beings create meanings based on their experiences to understand phenomena and answer ‘why and how’ questions (Busetto, Wick & Gumbinger 2020:14).

### Setting

The private hospital under study had two general medical wards. The study was conducted in these two general medical wards of a chosen private hospital in Gauteng province, where nurses treated patients diagnosed with depression.

### Study population and sampling

The study population comprised all the registered nurses permanently employed in the general medical wards of the selected hospital. To be fair and inclusive, all registered nurses who were permanently employed in this ward were approached. A total of 12 registered nurses were working in the general medical wards during the period of the research study. The study employed total population sampling (Bhandari 2022:para. 4), a strategy in purposive sampling where the complete, cooperative and accessible population exhibiting certain characteristics is examined. The invitation to participate in the study was sent to all registered nurses managing patients with depression in a general medical ward. Ten nurses agreed to be interviewed; however, data saturation (Creswell & Creswell 2022:198) was reached by interview eight, evidenced by no fresh information or themes coming to the fore.

### Ethical considerations

Ethical clearance and permission to carry out the research study were granted by the College of Human Sciences Research Ethics Committee (2020-CHS-42094577), the Hospital Head Office Research Committee (UNIV-2021-0019) and the hospital manager. Data collection was delayed because of the COVID-19 pandemic. All relevant COVID-19 government and hospital guidelines, such as social distancing, wearing a mask, sanitising hands and the venue when data collection commenced, were followed. A participant information sheet containing all information relevant to the study and a consent form were sent to all registered nurses in the general medical wards. Registered nurses who gave written consent to participate in the study were given time to ask questions before signing the study consent form. The researcher ensured confidentiality and anonymity by reporting the findings without divulging participants’ identities. Participant codes were used instead of participants’ real names, for example, P1. All data collected were kept on a password-protected computer. The transcriber and co-coder signed a confidentiality agreement.

### Data collection

Data were collected between July and November 2021. During the data collection period, South Africa moved between lockdown levels from stage three from 26 July to 12 September 2021 to stage one in October 2021. The researcher observed all COVID-19 protocols. The first author conducted 10 in-depth, face-to-face interviews in 2021 (Creswell & Creswell 2022:203). Interviews were conducted in English, as all professional staff were fluent in the language. The first author previously worked in these words; therefore, she applied reflexivity and bracketing by putting aside preconceived ideas of the general nurses’ experiences managing patients diagnosed with depression. A debriefing interview was conducted at three intervals before and during the data collection. An interview protocol was developed to direct the activities and form the basis of the field notes or records during the interview. The first author did a pilot interview of the protocol with two agency registered nurses whom the hospital did not permanently employ. These interviews did not form part of the dataset as these nurses did not meet the inclusion criteria of being permanent staff members at the hospital.

Interviews were conducted outside of official working hours, for example, before or after shifts, so as not to compromise official working hours or a period that would be convenient for each participant. The interviews took place in the general medical ward’s meeting room. A sign ‘Please do not disturb, meeting in progress’ was placed outside the door.

No participant was pressured to participate and was free to withdraw or stop an interview. This would not have any negative consequences should this happen. If any participant experienced emotional or psychological distress, the affected person would be referred to an organisational health employee free of charge. The organisational health employee would then provide the participants with the initial level of support and schedule counselling sessions. This intervention was not needed in this study.

A grand tour open-ended question was asked: ‘How is it for you to manage patients diagnosed with depression in a general medical ward?’ This was followed by facilitative communication techniques such as probing, clarifying, summarising and reflecting. All interviews were audio-recorded with permission using a high-quality digital recorder. Interviews were transcribed verbatim by the first author, with field notes taken for backup. Interviews lasted approximately 45 min each.

### Data analysis

Both authors were involved in the data analysis, each coding individually before a consensus discussion was held. A fine transcription approach was used (Moore & Llompart 2017:410–411), where all transcriptions were transcribed verbatim. Data were then analysed using a thematic analysis process (Creswell & Poth 2018:183). The initial stage involved multiple readings of the dataset, cultivating an intimate familiarity with its contents. Simultaneously, a comprehensive set of initial analytical notes was compiled, further enriching our understanding of the data. We conducted a systematic coding exercise upon this solid groundwork, identifying striking features throughout the dataset. These codes served as foundational stones, helping to unearth potential themes and acting as a catchment for the emergent patterns embedded in the data. Post this, a crucial stage of our process commenced with a stringent review to ensure the robustness of the identified themes. This involved meticulous scrutiny at two levels, individually coded excerpts and the broader dataset, thereby evaluating the consistency and accuracy of our themes and fostering a reliable thematic structure. Following this, we embarked on an exhaustive process of refining, focusing on each theme and its related sub-themes. This stage demanded refining the themes, reshaping them to represent both the visible characteristics and the deeper implications concealed within the data. Finally, both researchers reconvened, carrying their independently formulated understanding of the data to a consensus discussion. This collaboration facilitated the consolidation of individual perspectives, ensuring an integrated interpretation that captured the breadth and depth of the dataset. The process provided a nuanced and robust data analysis, laying the groundwork for further discussion and interpretation.

### Trustworthiness

Lincoln and Guba’s (1985) criteria for trustworthiness were used. Credibility was ensured by prolonged engagement. The researcher spent 30 min with each participant to obtain consent to participate in the study, 30 min before the interviews to build trust, create rapport and understand the views of the registered nurses and 45 min during the interviews. Gaining diverse perspectives from participants ensured the richness of the data and a fuller representation of the data. The literature consulted also included various views and discourses. The participants examined, explored and checked the transcripts to see whether their experiences were accurately represented. No changes were required. The researcher kept a reflective diary. By bracketing aspects such as potential biases, views and experiences, the researcher ensured that her background, personal values and position in the general ward had limited influence on the study.

Dependability and confirmability were ensured through an audit trail involving tracking and recording all decisions made during the study. Both authors coded the data. A thick description of the methods clarified how the data were collected and analysed. A thick description of the research context and processes was provided for the study to be replicated to facilitate transferability.

## Results

All 10 participants were black females. The participants’ ages ranged from 29 to 51 years, with a mean age of 22 years. They had 3–7 years of work experience.

This study was conducted during the COVID-19 pandemic (2021) when the disease was severe, and the participants’ views possibly represented an acute reaction to an unexpected and unknown emerging crisis. One main theme emerged with two related sub-themes and codes (Table 1). Each will be discussed with verbatim quotes from participants, followed by a literature control as a discussion.

**TABLE 1:** Themes, categories and codes.

Theme	Sub-themes	Code
The COVID-19 pandemic had a bidirectional impact on patients admitted to a general ward and the nurses. This added to the complexity of managing these patients	Detecting depression on admission or during the hospital stay is a problematic experience	Lack of training
		Inadequate resources
	Compounding aspects in patient care	Social distancing and isolation: the impact on the nurse-patient relationship
		Death anxiety
		Lack of support from hospital management

Note: The COVID-19 pandemic had a bidirectional impact on patients admitted to a general ward with depression and the nurses. Detecting depression on admission or during the hospital stay is a problematic experience because of a lack of training for nurses and inadequate human and other resources. This added to the complexity of managing these patients. Social distancing and isolation influenced the nurse-patient relationship. Both the nurses and patients were concerned about their mortality resulting in death anxiety. Through all this, they experienced a lack of support from the hospital management.

## Theme one: The COVID-19 pandemic had a bidirectional impact on patients admitted to a general ward and nurses

Managing patients diagnosed with depression during COVID-19 complicated nursing care because of the bidirectional and complex interaction of physical ailments, mental distress and behavioural aspects. This dynamic impacted both patients and nurses.

### Sub-theme 1: Detecting depression on admission or during the hospital stay is a problematic experience

Managing patients with depression in a medical ward was problematic before the outbreak of COVID-19 and became more so during and after the pandemic. Patients were often admitted with ‘other’ health issues over and above COVID-19. Depression was only identified later when a patient displayed certain ‘deviant’ behaviours or admitted to taking medication for depression. The following representative quotes reflect this further.

‘It is difficult to detect that the patient has depression while admitted to a general medical ward.’ (Participant 4, Age 35, Female)

‘According to me, it is hard because normally, in the general medical ward, patients with depression come with another illness only to find out later that patient has depression.’ (Participant 7, Age 38, Female)

‘A patient will come due to uncontrolled blood pressure and not mention a history of depression on admission; after some time, a patient will display changes in the behaviour. When I assess deeper and do other investigations, the patient will reveal that he is taking medication for depression at home.’ (Participant 6, Age 43, Female)

#### Lack of training

The registered nurses were mostly qualified in general nursing science in a general medical ward and lacked specific training, such as psychiatric training, which further compounded matters. When patients present with ‘*strange*’ behaviours, the untrained nurse might be unable to explain or misinterpret the behaviour. The following representative quotes provide further insight into this.

‘As a general nurse, I find it difficult to manage patients with depression in a general medical ward because I am not psychiatrically trained, and I treat them as normal medical patient patients only to find them with strange behaviour, such as when a patient cut himself with a knife and a fork from her lunch food tray when we dug and followed up the history, we found out that the patient was a psychiatric patient, but we nursed the patient like any patient with a general medical condition. I think if we can be trained, it might be easier for us to manage such patients.’ (Participant 2, Age 49, Female)

The work climate became stressful when the registered nurses lacked the knowledge and skills to manage patients diagnosed with depression in a general medical ward. Most participants highlighted the need for psychiatric training in the form of a specific course or in-service training.

‘A course in managing patients with depression and confusion in a general medical ward is needed, in this medical ward, especially for the registered nurses.’ (Participant 8, Age 29, Female)

‘Yes, I must be sharpened. So, I think the management should organise a program whereby nurses are equipped for the management of patients with depression in a general medical ward.’ (Participant 5, Age 28, Female)

‘A course in managing patients with depression and confusion in a general medical ward is needed, in this medical ward, especially for registered nurses.’ (Participant 3, Age 33, Female)

#### Inadequate resources

Detecting depression on admission or during the hospital stay is a problematic experience, often exacerbated by the lack of resources such as enough skilled nursing staff. The shortage of nurses in a general medical ward could be associated with the poor quality of nursing care. The participants stated that the staff shortage limits them from rendering care in an integrated nursing approach. Patients diagnosed with depression in a general medical ward are placed among patients with physical illnesses; a staff shortage prevents the registered nurses from giving these patients the special attention they deserve. This is further illustrated by the following representative quote.

‘In nursing, a comprehensive approach is demanded, but how will one be able to give an integrated approach if there is a staff shortage? Sometimes you need to address other patients, while a depressed patient needs your attention. There is often not enough attention given to the patients diagnosed with depression by the registered nurses because there is mostly one registered nurse in a shift and there are other patients that get sicker and resuscitated.’ (Participant 3, Age 33, Female)

The participants verbalised that managing patients with depression on a one-nurse-per-patient allocation ratio can help, creating greater availability of a nurse at the bedside. The following quote reflects this further.

‘So, it’s difficult, and at the same time, when we are short-staffed, we don’t even have time to spend with one patient and then try to get what is happening.’ (Participant 4, Age 35, Female)

Lacking personal protective equipment was an additional resource constraint. The following excerpts reflect this further.

‘Nursing COVID-19, as a nurse, I decided to face the challenges of my career, but I was not happy to find a shortage of personal protective equipment; I got discouraged first; we were told to keep a surgical mask on for seven days. The scrubs for attending to a patient were issued at first, but later we were told to purchase them ourselves because we were concerned about our health.’ (Participant 9, Age 34, Female)

### Sub-theme 2: Compounding aspects in patient care

Elements connected to social distancing and isolation added layers of complexity to the nurse-patient relationship. The looming concern about mortality shared by nurses and patients led to a pervasive sense of death anxiety. Amid these challenges, they felt a notable absence of support from the hospital administration.

#### Social distancing and isolation: The impact on the nurse-patient relationship

Because COVID-19 care was so demanding and the number of patients increased, nurses were advised not to spend too much time with each patient. Nurses feared nursing patients diagnosed with COVID-19, which created a further psychological divide. The decree not to allow hospital visitors created further hardship for the patients and affected their mental health. The following representative quotes and field notes reflect this further.

‘We were told not to take a long time at the patient’s bedside. If you are admitting a patient, do not take long. Just take 15 minutes to avoid standing a long time next to a very sick patient.’ (Participant 6, Age 43, Female)

‘This patient with COVID-19 and depression is what I saw, I am supposed to spend time sitting close to my patient, but because of fear of being infected and infecting my family, I was forced to spend little time at the bedside.’ (Participant 3, Age 33, Female)

The field notes also testified to this dynamic. The researcher observed that the multidisciplinary team members working under social distancing measures would talk to the patients while at the door, avoiding coming closer to the patient. Some patients advised nurses not to go near them because they would die. This also hampered vital care, such as physical examinations and assessments of patients.

Further social isolation occurred when no visitors were allowed during the earlier stages of the pandemic. The isolation caused additional distress to patients and nurses. The following quote demonstrates the matter.

‘Because number one, now they are hospitalised, there are no visitors allowed for a COVID-19 patient, so that in general is very depressing for a patient and me and on top of that, I can’t engage with the patient.’ (Participant 3, Age 33, Female)

Although fearful, nurses adapted and showed a positive attitude to caring for their patients.

‘I was also scared to nurse the COVID-19 patients at first but seeing that patients have no one else they have in this COVID-19 ward, seeing how desperate and some have lost hope, I picked myself up, and I was like I am going to nurse them with full compassion, and I am doing it.’ (Participant 1, Age 51, Female)

#### Death anxiety

The registered nurses in a general medical ward witnessed that COVID-19 was more dangerous than any other condition because it killed people, young and old. The registered nurses expressed the devastating state they found themselves working under and a desperate plea for support such as counselling. Different participants said it was strange to see young(er) patients dying in large numbers daily. One of the participants stated that they are used to patients recovering and not dying day by day. The following representative quotes provide further insight into this.

‘It was so devastating to see especially young people dying every day. You must lay the bodies every day. It was so difficult for us as nurses.’ (Participant 2, Age 49, Female)

‘But I think nurses needed psychological counselling during those early times because it was too much, it was too much. Every day is a corpse, every day laying a corpse, yet we are not used to those things. We are used to healing.’ (Participant 4, Age 35, Female)

‘So COVID-19 people know that COVID-19 is more dangerous than any other condition because it kills you; you can die in a short time.’ (Participant 5, Age 28, Female)

Some sick patients tried to protect nurses by telling them not to come to their rooms because they would succumb to the disease. They also did not want that the nurse should die. The following quote illustrates this.

‘Patients were also asking me, “sister, why are you coming to my room? I will die, and I don’t want you to die.”’ (Participant 6, Age 43, Female)

#### Lack of support from the hospital management

The participants mentioned increased responsibilities and expectations, yet they perceived a lack of support from management. Registered nurses were expected to be shift leaders and bedside nurses. The participants verbalised that there is less support from the hospital management in managing patients diagnosed with depression in a general medical ward. The registered nurses felt that the hospital management was only interested in their omissions and using their omissions against them. The registered nurses feared losing their jobs when they were told to write an incident report. This is further illustrated by the following representative quotes.

‘The patient was admitted with a drug overdose, took and broke a glass by hitting it on a bedside locker and cutting both her wrists and arms with that glass. When I, as the shift leader, notified the night manager at the beginning of the night shift, all the nurses were blamed for not taking good care of the patients. We were interrogated and threatened to lose our jobs.’ (Participant 10, Age 37, Female)

‘I understand that sometimes management sounds to be less supportive or understanding of the situation. We can stop lots of problems with patients with depression in a general medical ward if only management can listen to us as nurses on the ground.’ (Participant 8, Age 29, Female)

## Discussion

This section interprets the findings, provides links to previous research, addresses limitations and provides suggestions for future research.

The COVID-19 pandemic reciprocally affected patients admitted to general wards and the attending nurses, complicating patient management. Identification of depression at admission or during hospitalisation posed significant challenges. Hantrakul, Wangsomboonsiri and Sriphrapradang et al. (2020:23) concur with the study’s findings that while depression is common in a general medical ward, it remains problematic when it is not identified, detected or addressed. Munro and Milne (2020:19) substantiate this by noting that the detection and diagnosis of depression upon admission and during a patient’s stay in a general ward are often overlooked because of the similarity of its symptoms with those of other physical illnesses. For instance, physical conditions like hyperthyroidism also present symptoms such as mood swings, a poor attention span and fatigue.

Despite persistent efforts to train nurses in identifying depression, it remains inadequately diagnosed and treated globally and in South Africa (Kraus et al. 2019:127). Providing support to patients diagnosed with depression is crucial. However, some healthcare professionals lack competence in offering various types of support to these patients in a general medical ward. Borglin et al. (2019:4) advocate for the training of general nurses in supporting patients with depression and in assessing, implementing, monitoring and evaluating patient care holistically.

Nurses are vital for delivering compassionate care in all healthcare systems. Haddad, Annamararaju and Toney-Butler (2022:para. 2) agree that a shortage of nurses results in increased errors and mortality rates in hospitals. Suhaimi, Mulud and Sharoni (2021:75) express concern that a scarcity of nurses poses significant challenges in providing quality care in health systems. They, along with Suhaimi et al. (2021:78), highlight the worldwide issue of a nurse shortage, attributing it to global competition, an ageing population, and growing demands in healthcare delivery.

The COVID-19 pandemic exacerbated the depression epidemic in an already overwhelmed health system. Thaweerat, Pongpirul and Prasithsirikul (2021:20) also reported a significant prevalence of anxiety and depression in hospitalised COVID-19 patients, especially during peak periods of each wave. Shader (2020:962) expressed concerns regarding preventing a global COVID-19 depression, acknowledging the potential for a depression epidemic amid an already strained health system. Work-related factors such as staffing shortages, long working hours, increased workload and inadequate rest time were identified as key contributors to fatigue and psychological drain (Dall’Ora et al. 2020:15).

Similar to this study’s findings, Hugelius, Harada and Marutani (2021:3) agree that the restriction of visiting times in general hospitals contributed to feelings of loneliness, thereby increasing depression and aggression levels in patients diagnosed with depression.

In line with our findings, the limited availability of personal protective equipment (PPE) heightened nurses’ anxiety and fear (Karabağ Aydın & Fidan 2022:815). Suhaimi et al. (2021:75) reported that the fear and anxiety linked to death escalated during the pandemic. Nurses facing death anxiety may exhibit adverse reactions when caring for dying patients. Death anxiety has been implicated in the development of many mental disorders. During the COVID-19 pandemic, Mosheva et al. (2021:870) found that the death anxiety of nurses and patients often stemmed from witnessing the death of other patients of a similar or closer age. With patients dying away from their families because of contact restrictions and healthcare personnel operating without clear guidance, the fear and anxiety surrounding death were amplified (Yardley & Rolph 2020:para. 2). Khademi et al. (2021:350) agree that the high mortality rate of COVID-19, owing to the novel nature of the disease and limited scientific knowledge, escalated anxiety among nurses and patients alike. Death anxiety in nurses may be associated with depression, generalised anxiety and suicidal thoughts, potentially affecting their ability to care for patients (Moudi et al. 2017:48)

Patients diagnosed with depression often have concurrent physical illnesses, which impose additional demands on the already overburdened registered nurses. These nurses need psychological support to maintain their mental health stability, necessitating a fully staffed workforce.

During the COVID-19 crisis, nurses had to adapt on multiple fronts (Chau et al. 2021:8). It is recommended that both registered nurses and patients diagnosed with depression in a general medical ward benefit from a therapeutic nurse-patient relationship. This relationship plays a crucial role in enhancing patient feedback on nursing care, and it correlates with improved patient experiences and empirical outcomes (Cilluffo et al. 2022:1720). Research suggests that only nurses with strong determination can maintain effective communication and cooperation with patients while empathising at the bedside (Abu Sharour et al. 2022:4). Healthcare organisations should prioritise providing psychological support to all healthcare providers as a basic service (Catania et al. 2021:408).

Further research could explore the psychological adaptation of healthcare professionals during disasters to establish proactive measures for support and development in the future. The specific circumstances of the COVID-19 pandemic may limit the generalisability of the findings to non-pandemic times. The size and diversity of the nursing samples may limit the study’s conclusions. A larger, more diverse sample might yield different results.

## Conclusion

In conclusion, the complexities of detecting, diagnosing and treating patients diagnosed with depression within general medical wards were markedly compounded by the global COVID-19 pandemic. Our study underscores the vital role of trained nursing personnel in managing patients with depression, demonstrating the necessity of their adaptability in rapidly changing healthcare contexts. Concurrently, we identify the significant toll these responsibilities, coupled with a global health crisis, can take on nursing staff. A shortage of nurses, the fear and anxiety induced by the pandemic and the high demands of caring for patients with both physical and mental health conditions are factors contributing to the stress experienced by nurses. As a result, supporting nurses’ mental health is just as crucial as training them in holistic and effective patient care.
